# Vascular irregularities in COVID-19: findings on computed tomography pulmonary angiography

**DOI:** 10.1590/0100-3984.2024.0054-en

**Published:** 2024-11-18

**Authors:** Luiz Felipe Nobre, Sérgio Ferreira Alves Júnior, Elazir Barbosa Di Puglia, Rosana Rodrigues, Gláucia Zanetti, Edson Marchiori

**Affiliations:** 1 Universidade Federal de Santa Catarina (UFSC), Florianópolis, SC, Brazil; 2 Universidade Federal do Rio de Janeiro (UFRJ), Rio de Janeiro, RJ, Brazil; 3 Instituto D’Or de Pesquisa e Ensino, Rio de Janeiro, RJ, Brazil

**Keywords:** COVID-19, Virus diseases, Vascular diseases, Tomography, X-ray computed., COVID-19, Viroses, Doenças vasculares, Tomografia computadorizada por raios X.

## Abstract

**Objective:**

The purpose of this study was to evaluate the characteristics and meaning of the vessel wall irregularities sign, observed on computed tomography angiography of the pulmonary arteries of patients with coronavirus disease 2019 (COVID-19) pneumonia.

**Materials and Methods:**

This retrospective study of the computed tomography pulmonary angiography findings of 65 patients diagnosed with COVID-19 included 27 women and 38 men, with a median age of 52 years (range, 20-86 years). The diagnosis of COVID-19 was established through reverse transcription-polymerase chain reaction for infection with severe acute respiratory syndrome coronavirus 2.

**Results:**

The vessel wall irregularities sign was observed in 50 (76.9%) of the 65 patients with COVID-19. Among those 50 patients, the vascular involvement was predominantly mixed (arterial and venous) in 43 (86%), subsegmental in all 50 (100%), segmental in 13 (26%), bilateral in 46 (92%), affecting 4-5 lobes in 35 (70%), mainly in the right lower lobe in 46 (92%), and mainly in the left lower lobe in 44 (88%).

**Conclusion:**

The vessel wall irregularities is a prevalent sign of vascular involvement in patients with COVID-19.

## Abstract (pt)

**Objetivo:**

O objetivo deste trabalho foi avaliar as características e o significado do sinal das irregularidades parietais vasculares observado nas angiotomografias computadorizadas de artérias pulmonares de pacientes com pneumonia por COVID-19.

**Materiais e Métodos:**

Foram revisados, retrospectivamente, os achados angiotomográficos de 65 pacientes com diagnóstico de COVID-19. A amostra incluiu 27 mulheres e 38 homens, com idade entre 20 e 86 anos (mediana de 52 anos). O diagnóstico de COVID-19 foi estabelecido por meio de teste molecular RT-PCR para SARS-CoV-2.

**Resultados:**

O sinal das irregularidades parietais vasculares foi observado em 50 dos 65 pacientes com COVID-19 (76,9%), sendo predominantemente misto (arterial e venoso) (n = 43/50; 86%), subsegmentar (n = 50/50; 100%) e segmentar (n = 13/50; 26%), bilateral (n = 46/50; 92%), comprometendo de 4 ou 5 lobos (n = 35/50; 70%) e, principalmente, encontrado no lobo inferior direito (n = 46/50; 92%), seguido do lobo inferior esquerdo (n = 44/50; 88%).

**Conclusão:**

O sinal das irregularidades parietais vasculares é um sinal bastante frequente de comprometimento vascular em pacientes com COVID-19.

## INTRODUCTION

Since the onset of the coronavirus disease 2019 (COVID-19) pandemic, numerous studies have been conducted to evaluate imaging findings in pulmonary infections caused by the virus, more specifically with chest computed tomography. Bilateral ground-glass opacities, the crazy-paving pattern, and airspace consolidations with a predominantly peripheral and basal distribution have thus been established as the most common patterns found in patients diagnosed with COVID-19 pneumonia^**(1-4)**^.

In addition to the aspects of pneumonia caused by infection with severe acute respiratory syndrome coronavirus 2 (SARS-CoV-2), there is a second area of interest and research focus that is not yet fully defined: the prothrombotic state present in individuals with COVID-19^**(5)**^. Several case reports and some retrospective analyses have reported thrombotic complications in COVID-19, primarily pulmonary embolism, in a considerable proportion of patients^**(6,7)**^. On that basis, the associations that SARS-CoV-2 infection shows with pulmonary embolism, pulmonary microvascular thrombosis, disseminated intravascular coagulation, elevated D-dimer levels, and deep vein thrombosis (DVT) have been the focus of investigation and debate^**(6,8)**^. It is speculated that endothelial inflammation and hypoxic pulmonary vasoconstriction are the promoters of pulmonary thrombosis^**(5,9,10)**^. However, the pathological mechanism underlying pulmonary embolism/thrombosis in COVID-19 remains unclear. Even given the importance of this pandemic, detailed reports of pulmonary vascular morphology observed on computed tomography pulmonary angiography (CTPA) in COVID-19 are still scarce in the literature.

This study aimed to evaluate the characteristics and significance of one of the changes in pulmonary vascularization observed in patients with COVID-19 pneumonia-irregularities in the walls of the most peripheral vessels, arteries and veins, which we have designated the vessel wall irregularities (VWI) sign-and to determine whether there is a difference between the characteristics of that sign in COVID-19 and its characteristics in patients with pulmonary thromboembolism (PE) not infected with SARS-CoV-2.

## MATERIALS AND METHODS

This was an observational, retrospective cohort study involving 172 patients: 94 patients diagnosed with COVID-19 and with suspected PE; and 78 patients with a diagnosis of PE and without a diagnosis of COVID-19. All of the patients had undergone CTPA, and those images were evaluated. The patients with COVID-19 were evaluated at the hospitals operated by the Federal University of Santa Catarina and the Federal University of Rio de Janeiro (cohort). In contrast, those without COVID-19 were evaluated at the Rio de Janeiro branch of the Rede D’Or imaging center, with a specific protocol for PE. This study followed the current ethical standards for research involving humans, as well as ensuring the confidentiality and anonymity of the participant data. The project was approved by the Brazilian National Research Ethics Committee (Reference no. 29496920.8.0000.5262).

In both groups, the technical quality of the examinations was considered for selecting those appropriate for evaluation. Examinations in which there was no good contrast column (mean of 250 HU in the pulmonary artery trunk) were excluded. as were those in which there were movement or breathing artifacts and those that did not include the entire lung field in the Digital Imaging and Communications in Medicine images.

The CTPAs of the patients with COVID-19 were performed during the first wave of the pandemic (between February 2020 and November 2020). Of those 94 CTPAs, 29 were excluded because of unsatisfactory technical quality. Of the 78 CTPAs of the patients without COVID-19 (performed during the same period), 15 were excluded for that same reason. Therefore, the final sample comprised 65 CTPAs of patients with COVID-19 (COVID+ cohort) and 63 CTPAs of patients with PE without COVID-19 (COVID-/PE+ cohort). We finalized and calculated the statistical results from that sample.

The diagnosis of PE was based on the imaging findings on CTPA, the criteria for which have been established in the radiology literature^**(11)**^. The diagnosis of COVID-19 was based on the clinical history and the confirmation of infection with SARS-CoV-2 by reverse transcription-polymerase chain reaction, in accordance with existing guidelines^**(12-14)**^.

All of the examinations were performed in one of three 16-slice multidetector CT scanners (Activion 16; Toshiba, Tokyo, Japan), with a tube voltage of 120 kVp, automatic tube current modulation, pitch of 0.828, slice thickness of 1.0 mm, interslice gap of 0.8 mm, tube rotation of 0.5 s, standard filter, and programmed automatic triggering for contrast reading with a value of 80 HU in the pulmonary artery trunk, with caudal-cranial scanning during a breath hold with deep inspiration. Nonionic low-osmolar iodinated contrast (Omnipaque 300; GE Healthcare, Chicago, IL, USA) was infused intravenously with an injection system (Stellant D; Medrad, Warrendale, PA, USA) at a flow rate of 3-5 mL/s and a total infused volume of 70-100 mL, in the right arm if possible.

In addition to the conventional sequences, coronal and sagittal multiplanar reformatting was performed, and all CTPAs were evaluated with maximum intensity projection (MIP) between 30 and 40 mm. A pulmonary artery contrast enhancement threshold of at least 250 HU was considered ideal for case evaluation.

**Figure 1:**
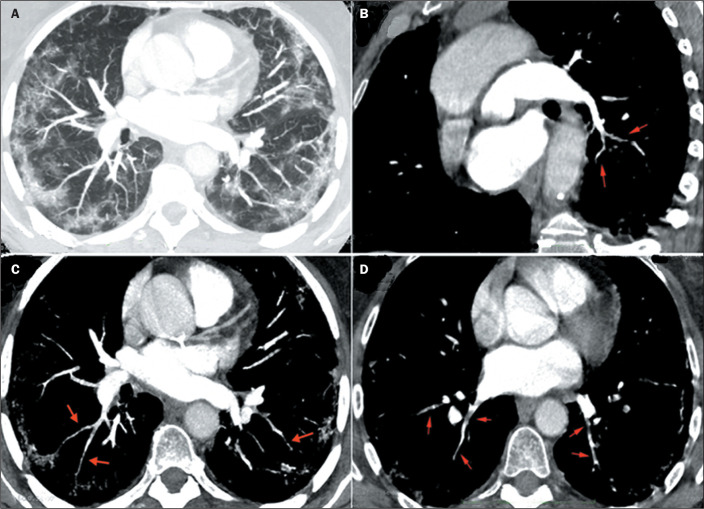
COVID-19 and signs of peripheral arterial and venous thrombosis. A: Peripheral ground-glass opacities. B: Filling defects in lobar arterial branches. C: Filling defects characterizing the VWI sign in small arterial branches in segmental and subsegmental areas. D: Filling defects and VWIs also observed in small pulmonary veins, on their path to the left atrium.

**Figure 2:**
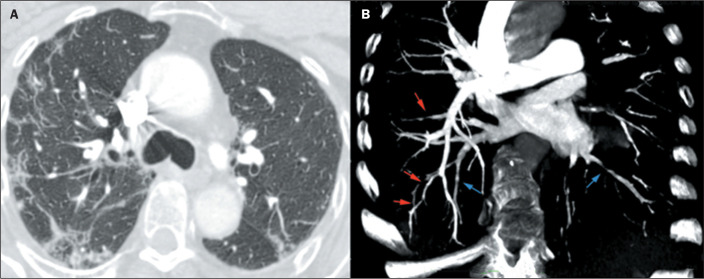
COVID-19. A: Peripheral and perilobular consolidations, together with ground-glass opacities. B: Filling defects in segmental and subsegmental arteries (red arrows), characterizing the VWI sign, also observed in peripheral veins in the lower lobes (blue arrows).

**Figure 3:**
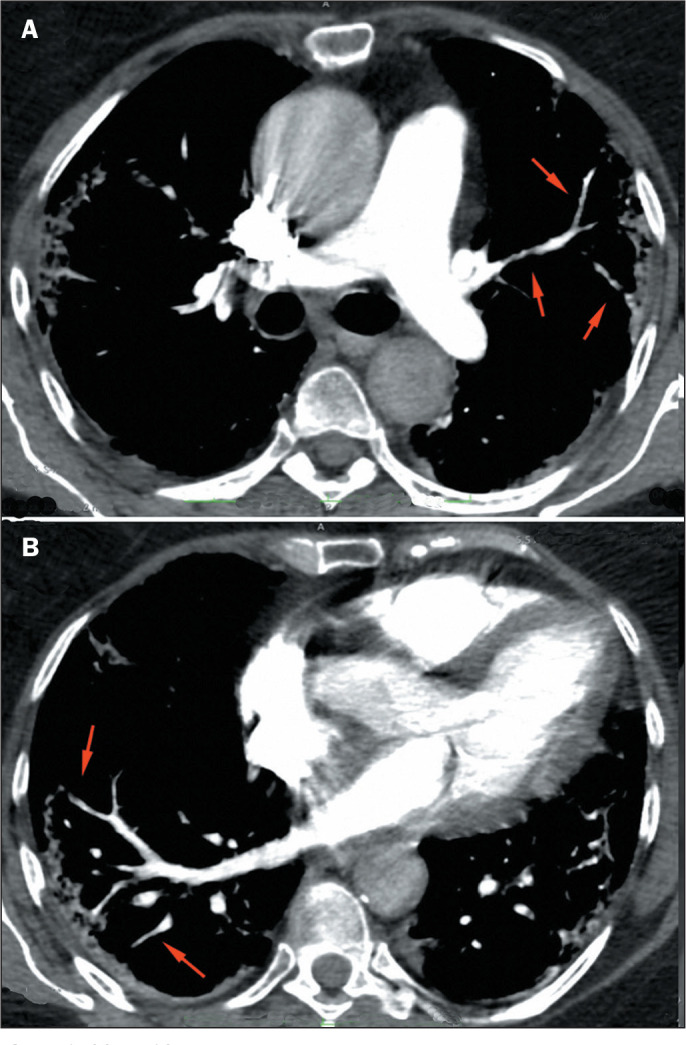
COVID-19. A: The VWI sign in segmental and subsegmental arteries (red arrows), associated with peripheral pulmonary opacities. B: Vascular irregularities are also observed in peripheral veins in the lower lobes.

**Figure 4:**
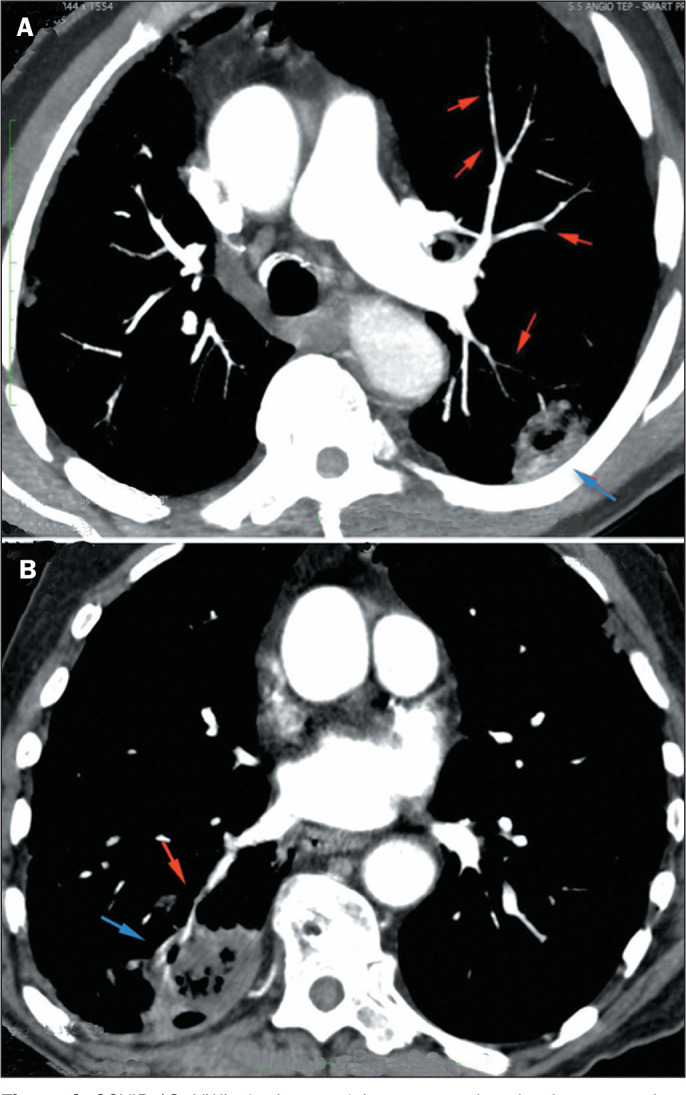
COVID-19. VWIs (red arrows) in segmental and subsegmental arteries (A) and peripheral veins (B), in different segments of both lungs, associated with cavitary peripheral consolidations (blue arrows), suggesting pulmonary infarction.

**Figure 5:**
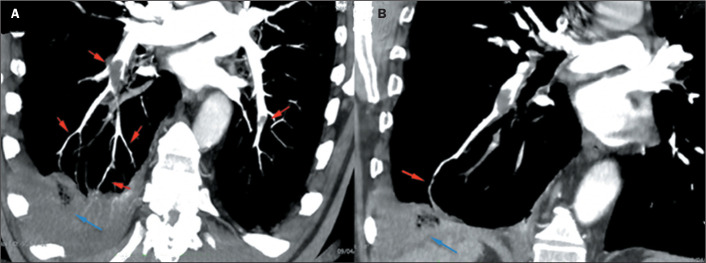
COVID-19. A: Filling defects in the right pulmonary artery and in small peripheral, segmental, and subsegmental branches, characterizing the VWI sign (red arrows). B: Filling defect in a small peripheral arterial branch in the right lower lobe (red arrow), in continuity with peripheral consolidation with a reversed halo sign with reticulation, characterizing pulmonary infarction, in addition to pleural effusion (blue arrows).

**Figure 6:**
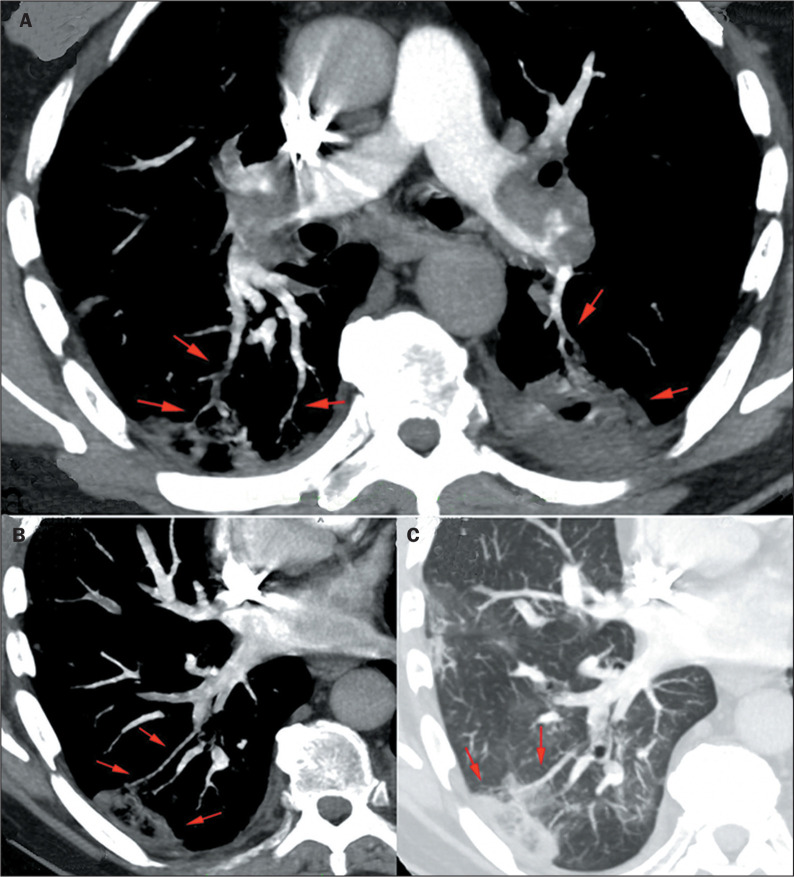
COVID-19. A: Filling defects in central pulmonary arteries and the VWI sign in small peripheral, segmental, and subsegmental branches (B,C), accompanied by peripheral wedge-shaped pulmonary consolidations, with a reversed halo sign with reticulation, characterizing pulmonary infarction (B,C).

We noted the presence of the VWI sign; the vascular sector affected (arterial, venous, or mixed); the location of the VWI sign (central, lobar, segmental, or subsegmental) and its laterality; and the number of lobes affected (1, 2-3, or 4-5). The VWI sign is a new image descriptor proposed in this study to differentiate between vascular manifestations associated with COVID-19 and those caused by PE inpatients without COVID-19. It is defined as changes in the walls of arteries, veins, or both (Figures 1 and 2), including single or multiple stenosed segments proximal to segments of normal or slightly enlarged caliber (Figures 3 and 4), and frequently associated with subpleural consolidations with characteristics of pulmonary infarction (Figures 4 to 6). Firstand second-order segmental or subsegmental involvement was considered distal, whereas central or lobar involvement was considered proximal. It is important to emphasize that the signal must necessarily be evaluated by MIP reformatting on a computer workstation. The evaluation of the original axial images alone does not allow the adequate study of irregularities in the caliber of blood vessels, especially small peripheral vessels, and it is precisely that lack that accounts for the underdiagnosis of thrombosis in small peripheral vessels in COVID-19. Therefore, it is recommended that MIP reconstruction should be routine in the analysis of patients with COVID-19, given that image viewing programs and workstations currently allow such reformatting to be performed. To increase the specificity of the finding in our study, the presence of the VWI sign was considered unequivocal and given weight in the statistical analysis only if it remained when the mediastinal window (300-400 HU and center level of 100-150 HU) was closed.

### Statistical analysis

Results are presented as absolute values and percentages for categorical variables and as medians (ranges) for numerical variables. Categorical variables were compared by using Fisher’s exact test or the chi-square test, as appropriate. Statistical analyses were performed with the IBM SPSS Statistics software package, version 26.0 (IBM Corp., Armonk, NY, USA), and Microsoft Excel 2019 with the Real Statistics Resource Pack. Values of *p* < 0.05 were considered statistically significant.

## RESULTS

Of the 65 patients with COVID-19, 27 (41.5%) were female and 38 (58.5%) were male. The median age was 52 years (range, 20-86 years). The VWI sign was observed in 50 (76.9%) of the patients with COVID-19. Of those 50 cases, 43 (86%) were of the mixed (arterial and venous) type, five (10%) were venous only, and two (4%) were arterial only. A total of 13 cases (26%) were segmental, and all 50 (100%) were subsegmental. There were no cases in which the VWI sign had a central or lobar location. The sum of the values is greater than 100% because it occurred in multiple locations in some cases. The VWI sign was bilateral in 46 cases (92%), unilateral on the right in two (4%), and unilateral on the left in two (4%). It was seen in one lobe in three cases (6%), in two or three lobes in 12 cases (24%), and in four or five lobes in 35 cases (70%).

Of the 63 patients in the COVID-/PE+ cohort, 43 (68.3%) were female and 20 (31.7%) were male. The median age was 38 years (range, 21-82 years). The VWI sign was observed in 17 cases (27.0%). In all of those cases, it was arterial only. The location was segmental in five cases (29.4%) and subsegmental in all 17 (100%). There were no cases in which the location was central or lobar. The sign was bilateral in 10 cases (58.8%), unilateral on the right in four (23.5%), and unilateral on the left in three (17.6%). It was seen in one lobe in six cases (35.3%) and in two or three lobes in the remaining 11 cases (64.7%).

COVID+ versus COVID-/PE+

The VWI sign was observed in 50 (76.9%) of the 65 patients in the COVID+ cohort and in 17 (26.9%) of the 63 patients in the COVID-/PE+ cohort. That difference was statistically significant (*p* < 0.001).

### COVID+/VWI+ versus COVID-/VWI+

The differences between the COVID+/VWI+ subgroup (n = 50) and the COVID-/VWI+ subgroup (n = 17) were statistically significant for the following variables: vascular sector affected-mixed in 86% of the COVID+/VWI+ subgroup patients and arterial in 100% of the COVID-/VWI+ subgroup patients (*p* < 0.001); laterality-bilateral in 92% of the COVID+/VWI+ subgroup patients and in 58.8% of the COVID-/VWI+ subgroup patients (*p* = 0.003); number of lobes affected-four or five lobes in 70% of the COVID+/VWI+ subgroup patients and in none of the COVID-/VWI+ subgroup patients (*p* < 0.001); and lobar distribution-left lower lobe in 88% of the COVID+/VWI+ subgroup patients and in 64.7% of the COVID-/VWI+ subgroup patients (*p* < 0.0??). There was no association related to the location of the VWI sign within the vascular network (*p* = 0.933).

## DISCUSSION

It is known that severe COVID-19 can be complicated by coagulopathy^**(15,16)**^. A thromboinflammatory state, associated with endothelial dysfunction, hypercoagulability, and activation of coagulation pathways, leads to an increased risk of microvascular and macrovascular thrombosis, which, in the most severe cases, manifests as disseminated intravascular coagulation, a prothrombotic state with a high risk of venous thromboembolism^**(16)**^.

The effect of SARS-CoV-2 infection on pulmonary coagulation and the consequent development of PE due to proinflammatory cytokines, endothelial dysfunction, and hypoxia are well established^**(17)**^. This assumption of a prothrombotic state inducing local thrombosis in COVID-19 pneumonia is supported by the association of PE with a greater extent of parenchymal changes, which suggests a connection to severe pulmonary inflammation beyond the common risk factors for PE or DVT^**(18)**^. That assumption is also supported by the findings of Suh et al.^**(19)**^ in a meta-analysis of 27 studies, which showed that DVT occurred in less than half (42.4%) of the patients with COVID-19 and PE.

Thrombotic complications related to COVID-19 include arterial and venous events, with microvascular thrombosis perhaps being the main contributor to the diffuse lung injury observed in patients who progress to respiratory failure and death, according to pathology studies^**(20)**^. In this form of prothrombotic pneumonitis, it is not yet known whether the mechanisms that promote microthrombosis are similar to those that promote large-vessel pulmonary embolic disease^**(20,21)**^. In investigating the role of pulmonary embolism versus that of local pulmonary thrombosis, some studies have suggested that thrombi detected by CTPA can, at least in part, be formed locally and as a consequence of thromboinflammation^**(22)**^.

In an autopsy study, Ackermann et al.^**(17)**^ reported that the microvascular architecture of the lungs differs significantly between patients with COVID-19 and those with influenza A (H1N1), in terms of microthrombosis and the density of vascular angiogenesis. Comparing seven lungs with COVID-19 and seven lungs with influenza A, the authors found that capillary microthrombi were nine times more prevalent in the lungs with COVID-19. The density of vascular angiogenesis by intussusception or sprouting was also significantly higher in those lungs. When observing the disposition of microthrombosis throughout the vascular bed, the authors reported microthrombi in precapillary arteries ≤ 2 mm in four of the seven lungs, without complete luminal obstruction, in alveolar capillaries in all patients and, to a lesser extent, in postcapillary venules. These descriptions can now be extrapolated to our imaging findings of the mixed VWI sign and heterogeneous venous contrast enhancement in some of our cases. Therefore, we believe that these findings are related to the presence of venous microthrombi and are a consequence, at least in part, of the distal pulmonary microthrombosis described in histopathological studies.

In another autopsy study, Menter et al.^**(23)**^, demonstrated microthrombi in the alveolar capillaries of five of 11 patients who died from COVID-19. Dolhnikoff et al.^**(24)**^ provided evidence to support that purported microangiopathy. From 10 lung autopsies of patients who died from COVID-19, obtained by ultrasound-guided transthoracic biopsy, the authors demonstrated diffuse exudative/proliferative alveolar damage, with intense epithelial viral cytopathic effect involving alveolar and bronchiolar cells, together with extensive small fibrin thrombi in small pulmonary arterioles. In addition, thrombi were found to be much more common in the lungs than in any other organ^**(5,25)**^. The fact that coagulopathy forms under prophylactic and systemic anticoagulation also strengthens the hypothesis of local clot formation^**(5,25)**^. It is therefore believed that most vascular thromboses develop locally.

It has been reported that the incidence of PE is elevated in severe acute respiratory syndrome^**(26)**^. In addition, peripheral microvascular thrombosis has been shown to be common in critically ill patients hospitalized with COVID-19, as demonstrated by Santo et al.^**(27)**^ in their study of 13 cases of thrombotic changes in the sublingual microcirculation of SARS-CoV-2-infected patients, in which the angiographic study revealed VWI secondary to partial or total luminal filling defects.

The most relevant angiographic finding of our study was the identification of irregularities in the walls of the most peripheral vessels, arteries and veins; that is, the VWI sign. As previously stated, 50 (76.9%) of the 65 patients with COVID-19, presented the VWI sign, compared with only 17 (27.0%) of the 63 patients with PE without COVID-19. That difference was statistically significant, allowing us to conclude that the presence of the VWI sign is associated with viral infection. Among the 50 patients with COVID-19 and the VWI sign, the predominant findings were mixed arterial/venous involvement (in 86%), subsegmental location (in 100%) segmental location (in 26%), bilaterality (in 92%), and involvement of four or five lobes (in 70%). In the 17 patients with the VWI sign without COVID-19, the predominant findings were arterial involvement (in 100%), subsegmental location (in 100%), segmental location (in 29.4%), bilaterality (in 58.8%), and involvement of two or three lobes (in 64.7%). When comparing the two subgroups, we detected statistically significant associations in relation to the vascular sector affected, laterality, and number of lung lobes affected.

The following morphological aspects of the VWI sign on CTPA were significantly associated with COVID-19: irregularities in the contours of arteries and veins (mixed involvement); bilateral occurrence; and involvement of two or more lung lobes. Similarly, the morphological aspects of the VWI sign on CTPA that were significantly associated with PE without COVID-19 were as follows: tortuosity of arteries only, unilateral occurrence, and involvement of only one lung lobe.

In the analysis performed by Mueller-Peltzer et al.^**(28)**^, 9 (56.3%) of 16 patients with respiratory failure due to COVID-19 and admitted to the intensive care unit presented thrombi in segmental and subsegmental pulmonary arteries. No thrombi were detected in segments with normal parenchyma, strengthening the local pulmonary arterial thrombosis hypothesis. The authors identified a significant association between pulmonary artery thrombosis and pulmonary parenchymal changes that were more severe, including more consolidations, suggesting the local formation of clots.

Cavagna et al.^**(18)**^ evaluated 101 patients with COVID-19 and identified PE in 41 (40.6%). Among those 41 cases, the PE was mostly bilateral or right-sided only (in 90.2%), mainly involving segmental arteries (in 90.2%) or subsegmental arteries (in 61.0%). Notably, DVT was present in only five (12.2%) of the 41 patients with PE, strengthening the proposition of local pulmonary thrombosis.

Pulmonary vascular irregularities in the presence of SARS-CoV-2 infection have been reported in a few previous articles, which opened the line of research on vascular lesions in 2020^**(17,29,30)**^. However, to our knowledge, no studies have evaluated the characteristics of such vascular irregularities.

Irregularity in the vascular contours of distal vessels (segmental and subsegmental), especially when mixed (arterial and venous), bilateral, and affecting the left lower lobe, constitutes the most prevalent vascular pattern on CTPA in COVID-19. When compared with the lungs of patients with vasculopathy due to PE of other causes, the finding of a statistically significant difference in all of the characteristics described supports and indicates a model of vasculopathy specific to SARS-CoV-2 infection.

Our study has some limitations. First, it was a retrospective study. In addition, because of the large number of institutions and collaborators involved, the CTPA images were acquired with various techniques. Furthermore, interobserver agreement was not assessed, which could have introduced an interpretation bias and variability in the evaluation of the results. Despite the limitations, we did not find any studies in the literature that focused on observation of the characteristics of irregularities in the contours of the pulmonary vasculature. That finding is unusual and indicates the existence of a peculiar pattern of vascular involvement.

Our study provides a starting point for new scientific research to expand the understanding of the imaging finding proposed here (the VWI sign) as a descriptor that indicates a disease pattern specific to infection with a coronavirus and its presence in the post-vaccination era.

## CONCLUSION

In this study, we examined the morphological characteristics of pulmonary vessels in 65 CTPAs of patients with COVID-19. The VWI sign occurred in 76.9% of our patients with COVID-19, affecting only distal (subsegmental and segmental) arteries, with a predominance of mixed (arterial and venous) involvement, occurring bilaterally and typically affecting four to five lung lobes. Our results add to the body of literature regarding pulmonary vascular involvement during COVID-19 and allow us to raise the hypothesis that microvascular thrombosis and the VWI sign are imaging findings that guide the diagnosis of infection by members of the coronavirus family.
